# Qualitative analysis of post-consumer and post-industrial waste via near-infrared, visual and induction identification with experimental sensor-based sorting setup

**DOI:** 10.1016/j.mex.2022.101686

**Published:** 2022-04-02

**Authors:** K. Friedrich, G. Koinig, R. Pomberger, D. Vollprecht

**Affiliations:** Chair of Waste Processing Technology and Waste Management, Department of Environmental and Energy Process Engineering, Montanuniversitaet Leoben, Franz Josef-Strasse 18, 8700 Leoben, Austria

**Keywords:** Sensor-based sorting, Identification model, Near-infrared sorting (NIR Sorting), Visual-spectroscopy sorting (VIS Sorting), Induction sorting, ALU, Arbitrary light units, AVAW, Chair for Waste Processing Technology and Waste Management, HDPE, High density polyethylene, HSB, Hue-saturation-brightness, HSI, Hyperspectral imaging, HSV, Hue-saturation-value, LDPE, Low density polyethylene, LLDPE, Linear low density polyethylene, MMI, Man-Machine-Interface, NIR, Near-infrared spectroscopy, PET, Polyethylene terephthalate, PLC, Programmable logic controller, PMMA, Polymethylmethacrylate, PP, Polypropylene, RDF, Refuse derived fuel, RGB, Red-green-blue, SBS, Sensor-based sorting, TPU, Thermoplastic polyurethane, VIS, Visual spectroscopy

## Abstract

Sensor-based sorting in waste management is a method to separate valuable material or contaminants from a waste stream. Depending on the separation property different types of sensors are used. Separation properties and their corresponding sensors are e.g. molecular composition with near-infrared sensors, colour with visual spectroscopy or colour line scan cameras, or electric conductivity with electromagnetic sensors.

The methods described in this paper deal with the development of **sorting models** for a specific **near-infrared, a visual spectroscopy and an induction sensor**. For near-infrared and visual spectroscopy software is required to create sorting models, while for induction only machine settings have to be adjusted and optimized for a specific sorting task. These sensors are installed in the **experimental sensor-based sorting setup** at the Chair of Waste Processing Technology and Waste Management located at the Montanuniversitaet Leoben. This sorting stand is a special designed machine for the university to make experiments on sensor-based sorting in lab scale. It can be used for a variety of waste streams depending on the grain size and the pre-conditioning for the sensor-based sorting machine. In detail the methods to create these sorting models are described and validated with plastic, glass and metal waste.

• Near-infrared spectroscopy measures the molecular composition of near-infrared-active particles.
• Visual spectroscopy measures the absorption of visible light by chemical compounds.
• Induction sensors use induced currents to detect nearby metal objects.

## Graphical abstract

**Figure:**
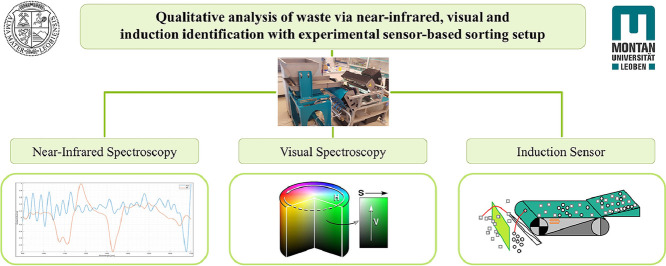

**Specifications table**

**Table:** 

Subject Area;	Environmental Science
More specific subject area;	*Sensor-based Sorting*
Method name;	*Qualitative analysis of post-consumer and post-industrial waste via near-infrared, visual and induction identification with experimental sensor-based sorting setup*
Name and reference of original method;	• *Near-Infrared Spectroscopy: Ozaki, Y.; Huck, C.; Tsuchikawa, S.; Engelsen, S.B. Near-Infrared Spectroscopy: Theory, Spectral Analysis, Instrumentation and Applications, 1st Edition, Springer, Singapore, 2021, ISBN: 978-981-15-8648-4.* • *Visual-Spectroscopy: Perkampus, H.-H. UV-VIS Spectroscopy and Its Applications, 1st Edition, Springer, Berlin, Heidelberg, 1992, ISBN: 978-3-642-77477-5.* • *Electromagnetic Induction: Morris, N.M. Electrical Principles II, 1st Edition, Palgrave, London, 1977, ISBN: 978-0-333-22062-7.*
Resource availability;	• *Hardware, Main Configuration: CLARITY Sorting System MONTANUNI-01, custom-made product constructed by Binder+Co AG* • *Software, Control Cabinet: M*a*n-Machine-Interface (MMI) by Binder+Co AG* • *Hardware, Near-Infrared Technology: EVK HELIOS NIR G2-320 by EVK DI Kerschhaggl GmbH* • *Software, Near-Infrared Technology: EVK Helios Optimizer; Version 3.4.2017.1* by EVK DI Kerschhaggl GmbH*, 08-2017 - Hardware, Induction Sensor: MESEP FS3 by Pulsotronic Anlagentechnik GmbH* • *Hardware, Visual Spectroscopy: AViiVA® SC2 CL Camera Link® Color Linescan Camera by e2v* • *Software, Visual Spectroscopy: FraunhoferICC by Fraunhofer IOSB, Version 2.5.0.0 by Fraunhofer IOSB, 2012*

## Method details

**Fig. 1:**
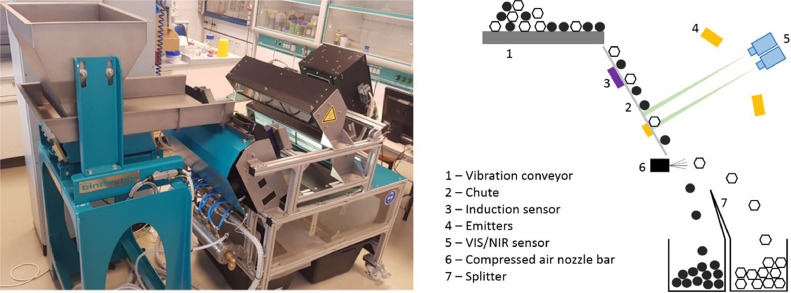
Functional schematic of the experimental sensor-based sorting setup at AVAW [3].

Sensor-based sorting is used in waste management for sorting and analysing waste streams and bulk materials. It is a non-contact, non-destructive process that offers a great deal of flexibility to cope with a wide variety of tasks. The Chair for Waste Processing Technology and Waste Management (AVAW) has an experimental sensor-based sorting setup for university and industrial research projects designed as a two-way machine. A grain size range from 5 to 300 mm can be processed. The feed takes place via a vibrating conveyor (1) followed by a glass chute (2) (see Fig. 1). The experimental sensor-based sorting (SBS) setup contains three sensors (referred to Fig. 1) that can be used for different waste streams:

- Near-infrared sensor (NIR) (5): waste glass, paper and cardboard, plastics, electronic scrap as well as construction and demolition waste.
- High-resolution colour line scan camera with the measurement principle of visual spectroscopy (VIS) (5): plastics, wood, paper and cardboard, waste glass as well as construction and demolition waste.
- Electromagnetic induction sensor (3): electric conductors, e.g. metallic waste.

It is also possible to combine several sensors to solve complex tasks with so-called sensor fusion.

Currently, norms are existing how to interpret NIR spectra with standard test methods like ASTM D 1925 Determination Yellowness Index or ASTM D 1003 Haze and Luminous Transmittance of Transparent Plastics, but none how to record all the data (VIS, NIR, induction, senor fusion) for such a setup, which is the focused method in this research paper [1,2].

In order to reproduce all applicable methods with the experimental SBS setup, the specifications of the sensors are listed. The first of the sensors used for classification via NIR Spectroscopy is the EVK Helios NIR G2-320, a high-speed hyperspectral imaging system. The main specifications of the EVK Helios NIR G2-320 are listed in Table 1.

**Table 1:** Technical Parameters of the EVK Helios NIR G2-320 Near Infrared Sensor.

Technical Data	Value
Spectral Range	930 – 1700 nm
Scan Rate	500 Hz full frame
Spectra Resolution	9 nm
Spectral Sampling	3.1 nm
Spatial Resolution	312 Pixels
Pixel Size	30 × 30 μm
Optical Coupling	C-mount lens
Slit	100 μm (80μm optionally)
Interfaces	GigE Vision, CamLink 2
Trigger Input	RS-485

The second sensor in application for the separation and classification trials conducted with the SBS setup explained above is the sensor for visual spectroscopy, the AViiVA® SC2 CL Camera Link® Color Linescan Camera. In the following, the essential key specifics of the sensor are depicted. The main specifications of the EVK Helios NIR G2-320 are listed in Table 2.

**Table 2:** Key Technical Properties of the AViiVA® SC2 CL Camera Link® Color Linescan Camera VIS Sensor.

Technical Data	Value		
**Sensor Characteristics at Maximum Pixel Rate**			
Resolution	1365 Red-Green-Blue (RGB) patterns or 4096 pixels		
Pixel pitch	10 µm		
Maximum line rate	14 kHz		
Anti-blooming	X 100		
**Radiometric Performances (Maximum Pixel Rate, T_amb_ = 25°C)**			
Output Format	12 bits (also configurable in 8 bit or 10 bit)		
Linearity (G = 0)	< 2 %		
Gain range (steps of 0.035 dB)	G_min_ -2 dB	G_nom_ 0 dB	G_max_ 22 dB
Peak response (1)(2) Blue Green Red	16.6 LSB/(nJ/cm²) 24.4 LSB/(nJ/cm²) 31.3 LSB/(nJ/cm²)	21.5 LSB/(nJ/cm²) 31.5 LSB/(nJ/cm²) 41 LSB/(nJ/cm²)	263 LSB/(nJ/cm²) 383 LSB/(nJ/cm²) 496 LSB/(nJ/cm²)
Dynamic Range	66 dB	64 dB	42 dB
Photo Response Non-Uniformity	± 4 % (± 15 % max)		

The third sensor used during trials at the sensor-based sorting stand is an induction-based sensor to detect metallic objects. It delivers a sensitive and accurate detection of small metal fragments. It delivers the detection results in real-time via Ethernet to a PC or a programmable logic controller (PLC), where the data can be evaluated. This way, the sensor's data can be coupled with the data delivered by other sensors like the NIR or VIS sensor to achieve complex sorting tasks. The main specifications of the Induction Sensor MESEP FS3 are listed in Table 3.

**Table 3:** Technical Properties of the Induction Sensor MESEP FS3.

Technical Data	Value
Interface	Ethernet RJ45; 10/100Mbit, RS485; 57.600 - 6.000.000 Baud; CAN; EtherCAT**
Sample rate	1 kHz
Resolution	12 - 100 mm
Protocol	UDP; HTTP(Ethernet); ASCII(RS485)
Number of Channels	4 - 124

Since correct illumination is vital for the detection with NIR, a halogen lamp is employed since halogen lamps deliver a flat spectrum in the NIR range. This specific illumination device, the Helen Dr. Fischer 15026Z with reflector, delivers a maximum illumination output in the detection area of 6.5 mW/mm² and is adjustable. It means the illumination setting allows dimming the lamp.

The complete data sheets of all employed sensors are found in the chapter “Additional Information” for further reference.

Tasks and applications that have been worked on in research projects on the experimental SBS setup are:

• Sample characterisation and determination of the composition,
• Creation of a digital grain size distribution,
• Discharge of contaminants,
• Enrichment of valuable substances,
• Sorting of bulk goods according to substance groups and
• Validation of sorting/separation results.

All of these tasks require the same method of qualitative analysis for sensor-based sorting, but the objective of the task is different.

### Requirements to get respectively good sorting results

Sorting results are influenced by internal and external factors, which have an impact on the process control. The internal factors are based on the construction of the sensor-based sorting setup, adjustments and settings on the machine:

• Belt velocity: throughput rate, relative velocity
• Air pressure: to blow out objects according to the sensor signal with the compressed air nozzle bar
• Valve diameter: influences the compressed air flow rate through one valve
• Valve distance: defines possible grain ranges to be sorted
• Splitter position: influences the sorting because of object weight and flight characteristics
• Position of the compressed air nozzle bar: influences the sorting because of object weight and ejection trajectories

The external factors which influence the sorting result are based on the properties of the material stream to be sorted:

• Grain size distribution: should be between 3 to 4 referred to the smallest and the biggest object in the fraction.
• Content of valuable material: the more valuable material in the input, the lower the influence of object overlapping.
• Grain form: agglomerates or objects, which are deformed, influence the sorting result either positively or negatively.
• Area density: too low can lead to incorrect sorting because of bad flight characteristics and too high can lead to incorrect sorting because the air pressure is not able to push the object over the splitter and into the reject fraction.
• Dust or steam between sensors and emitters can influence the identification of objects negatively; either objects are not identified, not recognised or the dust/steam cloud is identified as an object, which leads to an incorrect sorting result.
• Surface contamination: contaminations on the objects can cause that objects are incorrectly identified and wrongly sorted.
• Reflective surfaces: influence the transfer of the sensors light beam, it can cause positive or negative effects in the sorting result depending on the application. Positive: Reflective bands behind the specimen can enable measurement in transflection. Negative: Reflective materials can cause direct reflection into the sensor´s lense, causing misclassification or since direct reflection cannot be used by the NIR detector.

Further parameters, which influence the sorting result, can be set up on the man-machine-interface (MMI). For the correct identification of various materials, the correct calibration of the illumination is necessary. This is achieved by three illumination parameters in the MMI, namely the background light, in incident light and the intensity of the NIR emitters. These parameters can be set in a range of 0 - 100, corresponding with the percentage of the maximum intensity.

The background light is used for detecting the particles for ejection. Decreasing the background illumination can allow for the ejection of transmissive materials such as glass or clear PET bottles. This is necessary since excessive intensity may cause these materials to be ignored since the high intensity does not cause sufficient shadows for them to be identified. The background illumination should not be set higher than 20 %, because this leads to an overexposure of light which results in incorrect material identification.

Similarly, the identification for the VIS sensor can benefit from manipulation the incident illumination intensity in correlation to the surface properties of the material. Materials which absorb light very well may need a higher intensity than reflective materials whose glare can become an issue with excessive illumination.

The third illumination source to be calibrated is the NIR emitters intensity. Here a similar problem arises. Distinct materials can cause glare when illuminated with sufficient intensity, e.g. smooth PS containers. Here a reduction in NIR intensity can improve classification. Other materials with worse reflective properties, e.g. thin foils and multi-layered plastic packaging materials, benefit from increased NIR intensity. The reason for this is their thin material thickness, which limits the amount of radiation that can be reflected. With thin materials like plastic packaging foils most of the radiation emitted by the NIR illumination is lost to transmission because of the low material thickness. An increase in emission intensity can increase the overall radiation arriving at the specimen and therefore increase the amount of radiation reflected by the material, overall improving the detectability of these materials.

Some sorting tasks require the prioritisation of distinct materials over others present in the waste stream. In these applications, purity takes priority over yield. Here, the ejection of a particle that might be contaminated of wrongly classified is treated as more severe than the loss of a valuable particle. To achieve this prioritisation the sorting software allows for a weighing of material class pixels. This allows the user to multiply material pixels in the detection. Through this, the number of pixels of a contaminant might be counted tenfold, therefore ensuring the ejection of a contaminated or misclassified particle or an agglomerate containing a valuable particle, that might otherwise be ejected, reducing the purity of the valuable fraction.

Further parameters which have to be optimized for maximized sorting efficiency are:

• Delay time [ms]: Defines the time from the sensors object detection to the activation of the valve and needs to be set up so that the sorted objects can be blown out efficiently. It is mainly depended on the sorted objects weight.
• Minimum blow-out time [ms]: Defines how long the valve are minimum opened
• Minimum object width [mm]: Defines the minimum width of an objects, it can be set from 1 to 100 mm
• Valve activity [%]: Defines how far an object has to reach in a path so that the associated valve is activated, it can be set from 10 to 100 %.

In order to understand the principle for the explained methods in sensor-based sorting there are some definitions and statistics, that are mandatory to be understood:

• Pixel: A pixel is the smallest unit of recognition by the detector, determined by the detector´s resolution. These pixels make up the spectral image and are the basis for spectral evaluation. Each pixel contains information about its location and the intensities inherent at its location in all evaluated wavelengths. With this information each pixel is assigned a material class, which is then used to create objects for separation.
• Object: An object is a cluster of pixels. Whether an object is assigned to material class A or material class B is determined by the abundance of pixels making up the object. E.g. if an object consists of 49 % pixels classified as A and 51 % pixels classified as B, then the object is assigned to material class B. For separation purposes only, the objects classification is considered, therefore correct weighing of material classes is important in order to achieve a given sorting task at hand.
• Pixel statistics: Pixel statistics are the distribution of pixels between material classes, i.e. if an object consists of 50 pixels of material class A and 8 pixels of material class B, these proportions are assigned to the relevant classes separately.
• Material statistics: Material statistics are the classification of objects according to the dominant material class, e.g. if an object consists of 50 pixels of material class A and 8 pixels of material class B, the object is evaluated as material class A and all pixels (here 58 pixels) are assigned to material class A.
• Object statistics: The object statistics are the distribution of objects between material classes. If 58 pixels of class A are assigned to the object in the material statistics, this object is counted in material A´s object statistic, raising the object count by one.

The most crucial parameter in terms of plant settings from an operator's point of view is the throughput-rate $\dot{m}$. This parameter influences the economic performance of the sorting plant. The throughput rate determines the amount of material passing the experimental SBS setup during a specific time. The chute has a width of 0.5 m. The following formula is used to calculate the **throughput-rate** $\dot{m}$ [4]:

$$\dot{m}\left[\frac{kg}{h*m}\right]=\frac{m_{input}\;\left[kg\right]}{t\;\left[s\right]/3600\left[\frac{s}{h}\right]*0.5\;\left[m\right]}$$

where $m_{input}$ is the mass of the input material, and t the time of the sorting experiment. Additionally, four quality parameters should be determined to evaluate the performance of the sorting trial [4]:

• The **purity** is the quality of the product fraction (ejected fraction) and is calculated according to the following formula:
  $$Purity\;\left[\%\right]=\frac{m_{target\;fraction,\;eject}\left[kg\right]}{m_{\;eject}\left[kg\right]}*100\%$$
• The **yield** determines the efficiency of the ejection process and is calculated according to the following formula:
  $$Yield\;\left[\%\right]=\frac{m_{eject}\left[kg\right]*\;c_{target\;fraction,\;eject}\left[\%\right]}{m_{input}\left[kg\right]*\;c_{target\;fraction,\;input}\left[\%\right]}*100\%$$
• **Recovery** is the mass of ejected material relative to the mass of input material and calculated according to the following formula:
  $$Recovery\;\left[\%\right]=\frac{m_{eject}\left[kg\right]}{m_{\;input}\left[kg\right]}*100\%$$
• **Incorrect discharges** are material pieces, which are wrongfully ejected and their share is calculated according to the following formula:
  $$Incorrect\;\left[\%\right]=\frac{m_{eject}\left[kg\right]*\;c_{non-target\;fraction,\;eject}\left[\%\right]}{m_{input}\left[kg\right]*\;c_{non-target\;fraction,\;input}\left[\%\right]}*100\%$$

where *m*_input_ is the input mass, *t is* the time of the experiment, *m*_target fraction,eject_ is the mass of the target material in the ejected material, *m*_eject_ is the mass of ejected material, *c*_target fraction,eject_ is the percentage of the target material in the ejected material, *c*_target fraction,input_ is the percentage of the target material in the input material, *c*_non-target fraction,eject_ is the percentage of the non-target material in the ejected material and *c*_non-target fraction, input_ is the percentage of the non-target material in the input.

## Sorting with VIS technology

### Method principle

With the assistance of VIS-based sorting, materials can be sorted according to their colour. VIS-based sorting is the oldest sensor-based sorting technique, which was previously used mainly for waste glass. In polymer recycling, this sensor technology is often used for polyethylene terephthalate (PET). Its primary operating principles are well understood and thoroughly explained [5]. Nowadays, it is mainly used in combination with other sorting techniques.

The method is based on the interaction of electromagnetic radiation from the visible range (380 nm - 750 nm) with the sample. The colour of an object is determined by the absorption or reflectance of light in the visible range. The absorption of a specific wavelength is based on the excitation of valence electrons. The excitation of the valence electrons causes electron transitions between the energy orbitals of different energy. The resulting energy difference leads to the absorption of specific wavelengths according to the following equation:

$$\lambda =\;\frac{h\cdot c}{\Delta E}$$

where λ is the wavelength, E is the energy, h is the Planck constant (6,626*10^−34^ J*s) and c is the speed of light [6]. The smaller the energy difference, i.e. the easier it is to excite the electrons, the longer the wavelengths of light absorbed.

In the sorting process, the sample is exposed to electromagnetic radiation from a light source. Part of the light is absorbed and other parts are diffusely reflected on the surface of the object. These reflected parts are directed onto a detector. In this detector, the incident light is split into its components. The result is a spectrum of wavelengths as a function of intensity. With this sorting technique, only the range of visible light is analysed [7]. The colours can be defined either according to the red-green-blue (RGB) method or the hue-saturation-brightness (HSB) method. The RGB method defines the colour by parts of the primary colours red, green, and blue.

In comparison, the HSB method defines colour by hue saturation and brightness. The hue is displayed in a 360° circle representing a colour wheel, with each degree representing a specific colour. A saturation of 100% is the most intense version of the colour, regardless of the hue selected. In comparison, a saturation of 0 % represents the grey version of that colour. Brightness is also expressed as a percentage. A brightness of 0 % is black, no matter the hue or saturation. A brightness of 100 % means the light is at full strength [8].

With the VIS-based sorting technique, manual sorting by colour can be replaced. Compared to manual sorting, smaller grain sizes can be sorted with a higher throughput rate. The technique also enables the sorting of materials that are only slightly different in colour and would no longer be distinguishable by the eye (e.g. different shades of blue). However, successful sorting requires much preparatory work in defining the various colour classes and configuring the system correctly (lightning settings) [9]. The lighting settings must be adapted correctly, as they react very sensitively to external influences. When creating the colour classes, it must be ensured that no reflections occur in the picture of the reference material or that these are not considered when defining the colour class. The method works very well with materials that differ significantly in colour. When the colour differences are minor, the effort required to create the colour classes is very high [10,11].

Since materials vary in their ability to transmit visible light, the setup consists of two individual illumination arrangements. They are split up into two separate lighting modes, incident light and background light. Incident light is used with materials whose density does not permit light to be transmitted. The illumination source, therefore, needs to be on the same side as the detector. These materials include building materials like bricks which need to separate from the mortar according to their colour. The other category includes materials like glass, which are highly reflective and translucent. Their high reflectivity can inhibit the incident illumination by reflecting light directly into the detector lenses, causing glare. This glare can prohibit the detector from gathering sufficient information about the colour of the particle. Illuminating the particles from behind using background illumination can circumvent and alleviate these problems. Through background illumination, the particles tendency to cause glare is reduced and finer differentiation in the material's colour can be made. This allows good separation between different shades of the same colour, e.g. separating light blue glass from blue glass.

### Method description

**The first two steps** of a VIS sorting trial are typically adjusting the lighting settings and determining the white calibration and the black calibration to ensure optimal light that allows an equally good identification of the different colours and does not lead to overexposure.

White and black calibration aims to adjust and determine the spectroscope's colour response to a known colour composition under experimental circumstances like artificial light in the laboratory. It is done by taking an image of a standard colour before the experiments and calibrating the sensor's response. The object used for this is a white ceramic plate provided by the manufacturer specifically for this purpose. An image of this ceramic plate is taken which serves as a benchmark for what the sensor and the post-processing software regard as pure white or all detectable colours' similar composition in the visible wavelength range. Similarly, the black calibration is performed by shielding the detectors lense with a non-permissive plate, prohibiting stray light from entering the lens. This state sets the lower boundaries of brightness. These calibrations need to be performed before every measurement since changes in the ambient light due to changes in the daytime, weather and similar conditions can alter the colour of the specimen and render the prepared sorting model worse.

**Fig. 2:**
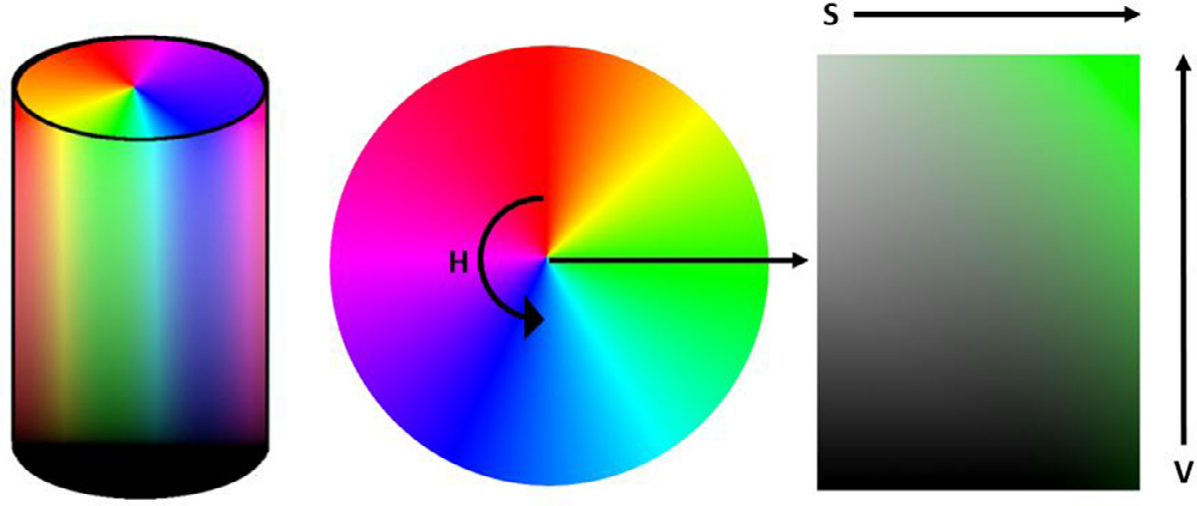
Colour cylinder for creating the VIS sorting model according to the colour sector (Hue, H), the brightness level (Value, V) and the saturation circle (Saturation, S) (authors depiction).

In order to separate and differentiate plastic parts by colour, the VIS sensor needs to be trained **in a third step** through creating a basic classification program. This program is developed by registering different colour types according to the Hue-saturation-value (HSV) system. Therefore, the HSV system settings need to be configured: The hue component, which represents the colour variations on a pie chart of 360°C, is divided into 48 units to differentiate between colours. The saturation parameter depicts the richness of the colour, and the brightness component defines how bright the colour is [8], as seen in Fig. 2. Both parameters are measured on a scale from 0 % to 100 % and divided into 200 saturation units and 250 brightness units, respectively. The more subdivisions into units of the HSV, the better is the resolution of the sorting trial, but the more complex and time-consuming the sorting system is capturing the different colours and training.

**Fourth**, some pieces of each chosen material – in the current example, a red low density polyethylene (LDPE), a white LDPE and a grey high density polyethylene (HDPE) – are inserted separately into the experimental SBS setup to picture the material. The picture allows the classification of the colour and consequently the detection and sorting using the VIS sensor for the sorting trial. The material stream, which goes through the chute, is classified by the colour of the targeted fraction in the HSV system in step **five**.

The software depicted and used to create the colour separation model is Teachin ICC. A teachin file defines the mapping of colours to specific classes. The file is read in by the sorting system so that during sorting it can be decided which colour classes are present for the pixels of an object detected in the camera image, in order to determine which material is to be assigned to the object based on the majority of colour classes present.

**Fig. 3:**
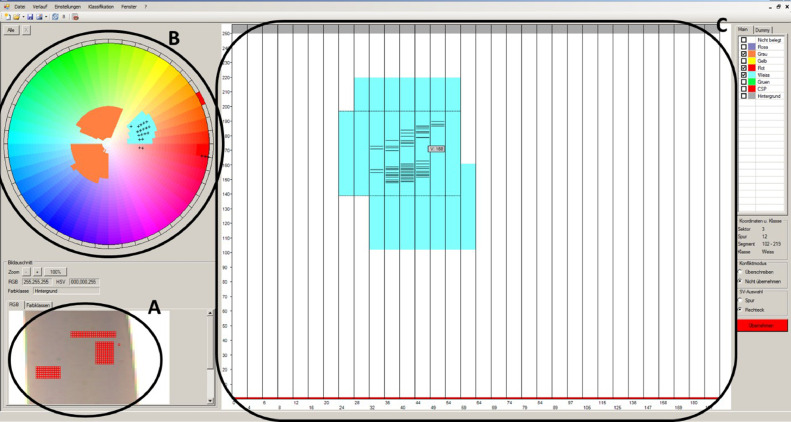
Creating the classification program in Teachin ICC by configuring the colour parameters according to the HSV system, here exemplary for the white LDPE material (authors depiction).

Fig. 3 shows exemplarily this procedure for the white LDPE fraction. After loading the picture (or a part of it) into the software Teachin ICC, a range of pixels is selected (A), which fits the material's colour. It shows little reflections and it is not situated at the edge of the material to avoid transparency. The hue pie then locates the colour in the respective segments (B). It also indicates other segments where previous materials have been localised. For example, the orange section in the pie represents the segments and saturation where the grey HDPE material is situated. By clicking on the different segments, the saturation (x-axis) and brightness (y-axis) diagram for the respective segment opens (C), showing where the corresponding area of the target material is located for the different selected pixels. In order to classify this range, the area is selected and saved for the respective colour. This procedure has to be repeated for all relevant hue segments (in the picture according to the segments, where the "x" is located).

**Fig. 4:**
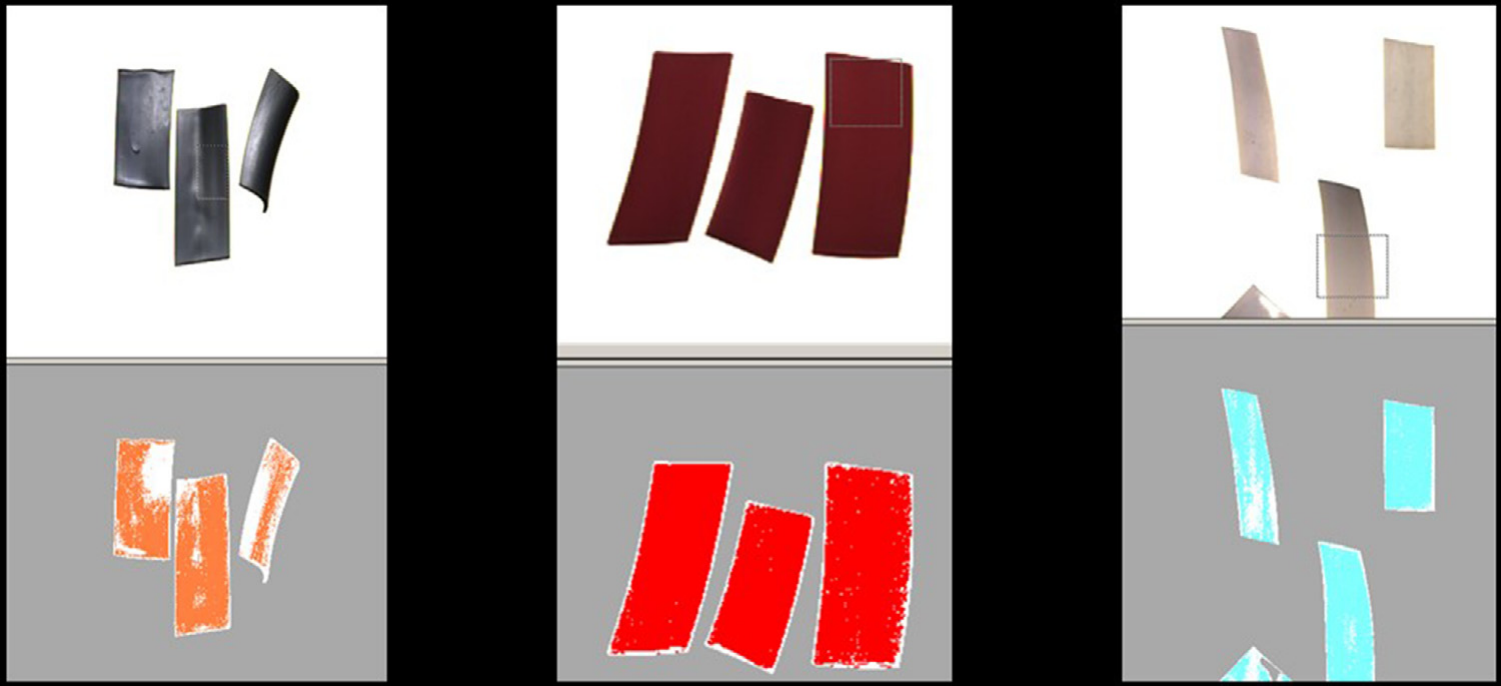
Verification of the coverage ratio for three different materials in Teachin ICC (left picture colour "grey" marked in orange, centred picture colour "red" marked in red, right picture colour "white" marked in turquoise) (authors depiction).

In order to have an effective classification, in a **sixth step,** it needs to be verified to what extent the selected HSV parameters can serve to detect and ultimately eject the targeted material by determining the coverage rate of the registered classification with the original picture of the material. These coverage ratios for three materials are visible in the following Fig. 4.

It is visible that the coverage ratio for red is optimal as it covers almost the whole surface of the three different material pieces. In contrast, the white and grey materials have a lower coverage ratio due to reflections and different exposure to the lighting system. The VIS sensor's inability to identify the material needs to be remedied. For this reason, the coverage ratio is optimised by adding more pixel and HSV ranges to the material classification in iterative procedure by repeating step five. Additionally, the parts that were successfully identified can be weighted with a higher factor. In this trial, grey and white are both being weighed twice as much as the other colours.

**Step seven:** After finishing the setup and configuration of the classification program, the program is transferred to the man-machine interface (MMI) of the experimental SBS setup and the target material for sorting is selected. The MMI is connected to the VIS sensor and ultimately controls the air nozzles that mechanically eject the selected material pieces through an air blast. The pressure applied as air blast from the valves in the air nozzle bar is defined, reflecting sufficient pressure to move the target material pieces over the splitter. The time delay between detection and ejection, Δt, is defined to consider the distance between the classification area on the chute and the air nozzle bar and reflect on the density and falling behaviour of the material. For the trials the white LDPE material is the target material for ejection.

In **step eight**, the actual sorting trial is conducted by inserting the test fraction into the experimental SBS setup, thus, putting it on the vibration conveyor and running the system. The pieces fall down the chute where the VIS sensor detects the targeted fraction, which activates a specific valve in the air nozzle bar according to the position of the targeted material piece. This airflow ultimately sorts the material detected as white over the splitter into the target box, whereas the non-target fraction falls into the reject box.

As the final and **ninth step**, the two sorted fractions are manually sorted and weighed per target and non-target material content to determine the performance parameters of the sorting trial.

### Method application

The feed material is a mixed fraction of different plastic components with a corresponding colour, see Table 4. A high-resolution line scan camera (VIS technology) is used as the sensor. The aim is the purest possible extraction of white material from the feed material. Following Fig. 1, the material is separated using a vibration conveyor (1) and moved into the sensor's exposure area via a slide or chute (2).

**Table 4:** Material and corresponding colour of the feed material components.

Material	Colour
LDPE	red
LDPE	white
LLDPE	green
HDPE	grey
PP	purple
TPU	yellow
PET	clear

This sensor assembly (4 - 5) consists of an emitter (4) and a detector (5). In this case, halogen lamps, fluorescent tubes, or LED strips are usually chosen as emitters. The emitter's radiation is partially reflected by the individual pieces of plastic and measured by the detector. The detector is connected to a computer that records the detected colour in the “colour cylinder”. In terms of the colour sector (Hue, H), the brightness level (Value, V) and the saturation circle (Saturation, S) in a previously created colour cylinder model (see Fig. 2) are compared and thereby assigned to a defined group.

According to the task, if a piece of plastic belonging to the “white” group is recognised, it must be separated from the remaining fragments. That is done using a compressed air blast. A valve strip (6) downstream of the sensor opens one or more valves when the white piece is in front of the valve strip. The piece is "shot out" over the separating edge (7). All different coloured plastics are deliberately not ejected.

### Method validation

**Fig. 5:**
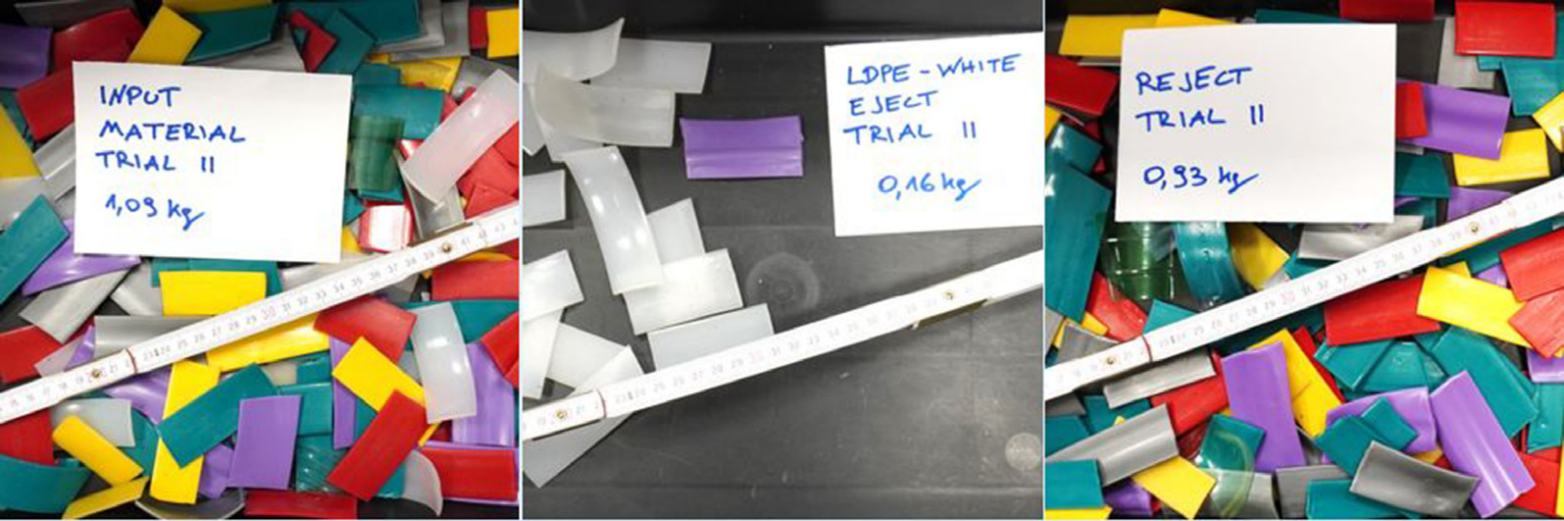
Feed material (left), separated white LDPE – Eject (centre) and coloured plastic – Reject (right) (Trial 2 in Table 5) (authors depiction).

The validation use-case is to separate “white” as target fraction from the feed material described in Table 4 and the left picture of Fig. 5.

Table 5 sums up the data from the trials in the VIS experiment. The white LDPE material was targeted for ejection in all sorting trials. Table 6 provides the consequent sorting trial results in terms of plant and quality performance parameters. The resulted fractions from the trial are shown in the centred and right picture of Fig. 5.

**Table 5:** Data of the VIS experiments.

	Unit	Trial 1	Trial 2	Trial 3	Trial 4	Trial 5	Trial 6	Trial 7	Trial 8	Trial 9	Trial 10
Time of experiment	*s*	34	38	42	42	42	40	42	41	40	38
Input mass	*kg*	1.09	1.09	1.09	1.09	1.09	1.09	1.09	1.09	1.09	1.09
Mass of eject	*kg*	0.16	0.16	0.18	0.17	0.17	0.16	0.15	0.17	0.15	0.17
Mass of reject	*kg*	0.93	0.93	0.91	0.92	0.92	0.93	0.93	0.92	0.93	0.92
Target material in eject	*kg*	0.15	0.15	0.16	0.16	0.17	0.16	0.14	0.16	0.14	0.16
Target material in reject	*kg*	0.02	0.02	0.01	0.01	0.00	0.01	0.02	0.01	0.03	0.01
Non-target material in eject	*kg*	0.01	0.01	0.02	0.01	0.00	0.01	0.01	0.01	0.02	0.01
Non-target material in reject	*kg*	0.91	0.92	0.90	0.91	0.92	0.91	0.92	0.91	0.91	0.91

**Table 6:** Results of the VIS experiments.

	Unit	Trial 1	Trial 2	Trial 3	Trial 4	Trial 5	Trial 6	Trial 7	Trial 8	Trial 9	Trial 10
Throughput-rate	kg/(h*m)	230,6	206,3	186,7	186,7	186,7	196,0	186,7	191,2	196,0	206,3
Purity	*%*	93.8	96.8	86.6	93.9	97.7	94.5	94.1	93.6	90.3	92.0
Yield	*%*	90.5	89.2	92.3	93.9	100.0	92.3	88.9	95.2	83.7	95.2
Recovery	*%*	14.9	14.1	16.4	15.2	15.8	15.1	14.0	15.7	14.1	16.0
Incorrect discharges	*%*	1.1	0.5	2.6	1.1	0.4	1.0	1.0	1.2	1.6	1.5

## Sorting with NIR technology

### Method principle

Nowadays, near-infrared (NIR) sorting systems are state-of-the-art in plastic waste sorting plants [4]. The basic working principles of NIR spectroscopy were the subject of a plethora of scientific studies, so they are well understood and can be used and modified to achieve a variety of tasks [12]. The NIR spectroscopy is based on the partial absorption of light in the NIR region (750 – 2500 nm) by the molecules in a material. Other than photons of UV and visible light, IR photons lead to vibrational and rotational movements of molecules or molecule parts. Suppose the frequency of the incident IR light correlates with the resonance frequency of molecular vibration. In that case, the IR light is absorbed, resulting in the molecule's or functional groups' vibrations. However, IR light can only be absorbed if the vibration changes the dipole moment in the molecule or functional group. By detecting the reflected or transmitted light of the irradiated material, absorption bands in specific spectral regions can be located. Based on these bands' position and intensity, the functional groups within the material and, therefore, the material itself can be identified [13].

Methods based on NIR spectroscopy are characterised by the fast, non-destructive and non-invasive principle. Additionally, they are more suitable for in-line use than the mid-range infrared systems because of their lower price and higher robustness [14,15].

The basic principle behind NIR systems in sorting plants is irradiating the objects with NIR light and detecting the reflected light by a sensor. For successful sorting, the system has to be trained beforehand with the spectra of different materials. The detected spectrum is then pre-processed, which entails normalisation and derivation to emphasize their specific characteristics. These processed spectra are then compared with the spectrum of the previously defined material to be ejected. If the similarity is high enough, the respective object is identified as the defined material and ejected. The similarity necessary for assigning the material to an existing class can be defined by the user via the threshold parameter in EVK SQALAR. In most cases, specific wavelength regions in the spectra are defined for comparison rather than the entire spectrum. In this way, the computing time can be reduced.

This sorting method also has its disadvantages as it is a binary sorting system that can only target one fraction to be sorted out. Thus, several NIR systems have to be connected in series or cascades to sort out multiple fractions. Another point that should be kept in mind is that moisture, dirt, or other residues can influence the NIR-spectra, leading to mis-sorting [4]. Furthermore, it is impossible to sort black or very dark plastics as they show high absorbance and, therefore, low reflectance [16]. An alternative to NIR sorting systems are tracer-based or water-mark sorting systems, which can sort a waste stream into several fractions in one step. Nevertheless, these two technologies also have their challenges, e.g. in technical feasibility and economic performance.

### Method description

Similar to the proceeding in the VIS experiment, the NIR sorting trial starts with classifying the different target materials for the program's configuration. As for the VIS experiment, the light settings and the white calibration and black calibration for the NIR experiment are set up for the sorting task.

Before a measurement can take place, the sensor's white and black calibration needs to be performed. The reasoning behind this calibration is that the software needs to know the maximum and minimum radiation intensity to expect, setting the upper and lower boundaries for spectral evaluation. The white calibration is performed as follows. Firstly, a reflective material is placed on the chute, a white ceramic plate provided by the manufacturer for this purpose. Then the user sets the white calibration target in the EVK SQALAR software, in this case, 2000 Arbitrary Light Units (ALU), which is the unit for radiation intensity used by EVK in all their software and detection applications. This target correlates to the reflected intensity by the ceramic plate. The software will use this calibration target as a reference to order the detected radiation according to its intensity. If any pixels' reflected radiation exceeds this threshold, its intensity will be capped to the white calibration setting.

After the white calibration has been performed, the black calibration follows. All incoming light into the detector must be blocked with a non-NIR permissive shielding, usually made from black polymers, coloured with carbon. Then the user starts the black calibration process in EVK SQALAR, defining the bottom threshold, 0 ALU, of incoming light. After both processes, black and white calibration, have been completed, the intensity range under the given experimental circumstances has been defined. This intensity range is used to plot and evaluate the spectral information of the evaluated materials.

In addition, the background´s reflection intensity needs to be defined in SQALAR. The glass chute (2 in Fig. 1) is transmissive, leading to low reflected intensity if no object is present to reflect the incident NIR radiation. This lack of reflection is exploited by defining a lower boundary of intensity under which all pixels are classified as background. All background pixels are omitted from classification.

**Fig. 6:**
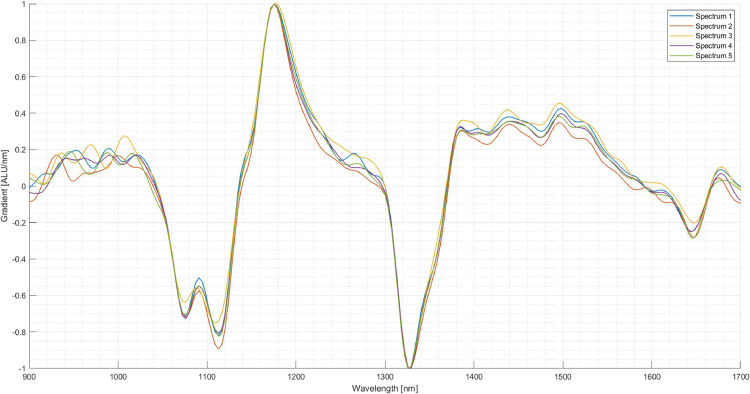
Scattering of the first derivative of the NIR-spectrum of PP amongst different Pixels (evaluation performed in MATLAB, authors depiction).

**Fig. 7:**
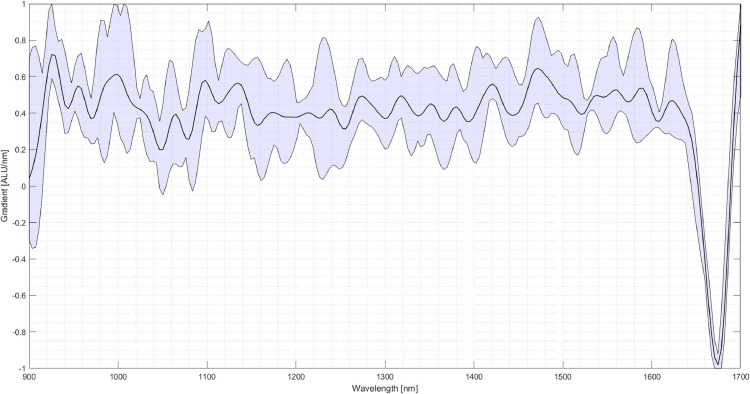
Scattering of the first derivative of the NIR-spectrum of PET (blue) (evaluation performed in MATLAB, authors depiction).

**Fig. 8:**
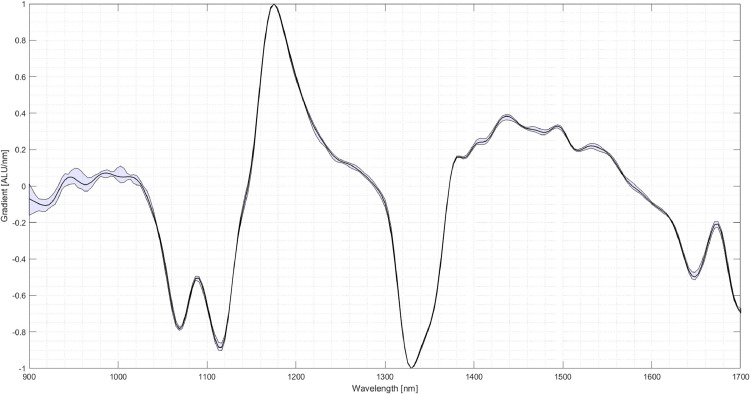
Scattering of the first derivative of the NIR-spectrum of yellow TPU (evaluation performed in MATLAB, authors depiction).

The next step is the setup of the system. In this case, three materials for creating the classification program are chosen: Polypropylene (PP), PET and thermoplastic polyurethane (TPU). Several pieces are taken and inserted into the experimental SBS setup to acquire an image and the corresponding NIR-spectra for each material. From the images of the pieces, several pixels are selected over which the respective NIR-spectrum is averaged. Fig. 6 shows the spectra of five selected pixels of a PP specimen. It can be seen that the spectra vary amongst those pixels although it is the same material. When selecting the areas of the particles, areas with reflections and edges should be avoided. The received spectrum is then assigned to the respective material. For the following comparison between the materials, the first derivative of the spectrum is used. The scattering of the spectra of the three materials is shown in Fig. 7, Fig. 8. As visible in the Fig.s, PET exhibits a relatively high scattering compared to the other two plastics. However, due to its characteristic peak in the area of 1650 nm, PET is usually easy to detect, especially in this case compared to PP and TPU.

The spectra can be evaluated using their depiction in a cartesian coordinate plane. The x-axis of this plane depicts the relevant wavelength in nanometres. This relevant wavelength represents the wavelengths the detector acquires, in this case, 930 nm - 1700 nm. This label does not change, regardless of the post-processing, the spectra undergo. The y-axis depicts the intensity of the reflected radiation acquired by the sensor. The y-axis' unit is the arbitrary light unit (ALU). As mentioned, this unit is used by all operating systems created by EVK and represents the detected intensity in relation to the white and black calibration. The range of this is set by the user or the manufacturer when setting the target for white calibration. In the case of this study, the white calibration target is set to 2000 ALU, representing the maximum intensity of the radiation reflected by the specimen used for white calibration. In this case, the background used for the calibration was a white ceramic plate supplied explicitly by the manufacturer for white calibration. As mentioned, the label of the x-axis does not change with progressing processing of the spectral data, e.g. derivation. It is not applicable for the y-axis, as its label changes with processing the spectral data, depicting the relevant information for the current processing application, e.g. the gradient of the raw spectra when displaying the first derivative. In this example, the unit of the y-axis changes to depict the change in intensity over the given wavelength, represented as arbitrary light units per nanometre (ALU/nm). However, this is not represented in the current version of the used classification software. The representation of the y-axis increases the range to permit the representation of the derivatives of the raw spectra. This part of the software can confuse when interpreting the spectra and needs to consider when preparing spectra for publication and use compared to other spectra, taken under different circumstances and with different levels of processing applied to them. With knowledge of this peculiarity in the analysis software, caused complications can be successfully circumnavigated. E.g., by using external software to analyse and compare spectra, a MATLAB script translates the raw spectral hyperspectral imaging (HSI) cube into a spectral image. Out of this cube, suitable spectra can be selected, processed, evaluated and plotted.

**Fig. 9:**
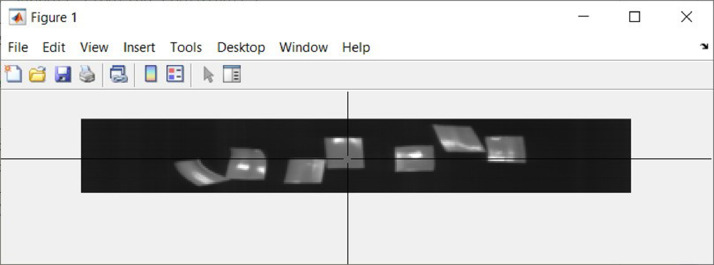
Selection of pixels for evaluation from the visualised HSI Cube in SQALAR (authors depiction).

The code used in the comparison of spectra takes the HSI Cube, exported as a .mat file. This HSI cube's dimensions represent the size of the spectral image taken and the number of spectral evaluation points linearly spaced over the relevant wavebands. In this case, the detector can assess 220 spectral points in the detectable range from 930 nm - 1700 nm. Therefore, the dimensions of this HSI cube are [Width of the spectral image in pixels x Height of the spectral image in pixels x 220]. The code converts this HSI Cube into a black and white image, representing the average reflected NIR intensity at every recorded pixel. This average produces an interpretable representation of the spectral information contained in the HSI Cube from which pixels for spectral evaluation can be selected. This selection process is depicted in Fig. 9, which shows the representation of a spectral recording taken of seven PP specimens.

**Fig. 10:**
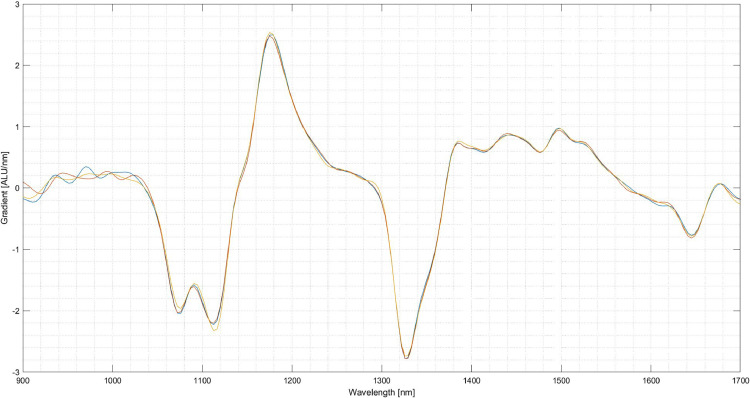
Spectral evaluation of PP pixels after processing (evaluation performed in MATLAB, authors depiction).

After selection, the user can process the spectral information as needed. For example, apply smoothing, normalisation and derivation, enabling the user to exert more control over the data processing. An example of three evaluated pixels from the spectral image mentioned above is depicted in Fig. 10. This Fig. depicts three PP spectra of the specimen after applying the first derivative, gaussian smoothing with a smoothing interval of 10 and normalisation using the z-score method, which normalises the data to have a mean of 0 and a standard deviation of 1. A smoothing interval of 10 means the smoothing was applied taking the median over a ten-element sliding window.

These spectra are used as labelled input for the machine learning algorithm underlying the spectral classification. These spectra serve as the training data for the supervised machine learning approaches used to label new spectra or in other words, to classify materials into pre-defined groups. In order to achieve this, partial least square regression is used. This approach allows the classification of material without the need to explicitly program every spectrum which could likely be encountered when sorting materials. The rigor, with which spectra which deviate from the training set are discarded, or counted as “not classified”, can be determined by the user via the previously mentioned threshold parameter.

**Fig. 11:**
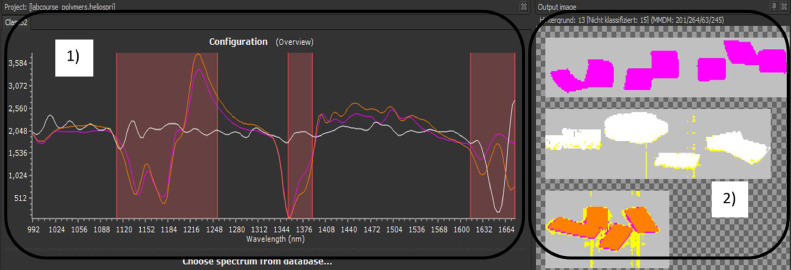
Creating the classification program in SQALAR for the NIR experiment: 1) selecting three wavelength sections visualised by the red areas, 2) coverage ratios of the used pieces (PP purple, PET white, TPU orange) (authors depiction).

Once the spectra have been successfully assigned to the materials, the wavelength range is selected in SQALAR for usage in the following sorting process to select specific ranges in which the spectra differ significantly. Fig. 11 shows the chosen wavelength ranges in the left side of the Figure (1). The images on the right side of the Figure (2) show the pieces and the classified material type, visualised by the respective colour. It is shown that PP is covered best. PET is also well covered, except for some small areas at the edges assigned to unclassified material (yellow). The yellow lines in the frames can be attributed to dirt on the chute. The third image shows TPU, which has larger misclassified edge areas identified either unclassified or PP. Since the coverage ratio is greatly exceeding 50%, sorting should be feasible. The higher weighting of the successfully identified parts can further improve the sorting.

After finishing the classification program and transferring it to the MMI, the settings of the air nozzles are adjusted as described previously for VIS technology. For the sorting experiment, PP is the target material for ejection.

The sorting trial is started by putting the test fraction on the conveyor and running the system. The principle of the sorting process is the same as for the VIS sorting. After the sorting is finished, the two separated fractions are manually sorted by target and non-target material and then weighed to evaluate the sorting process's quality.

### Method application

The feed material is a mixed fraction of different plastic components with a corresponding colour, see Table 4. NIR spectroscopy is used as sorting technology. The aim is to achieve the purest possible output of PP (following Fig. 1).

The material is separated using a vibration conveyor (1) and moved into the sensor's exposure area via a slide or chute (2).

Modern NIR sensors (5) cover a wavelength range from around 1000 to 2500 nm. Halogen lamps, for example, can be used as emitters (4). This spectrum contains information that allows conclusions about the chemical composition of the investigated objects.

**Fig. 12:**
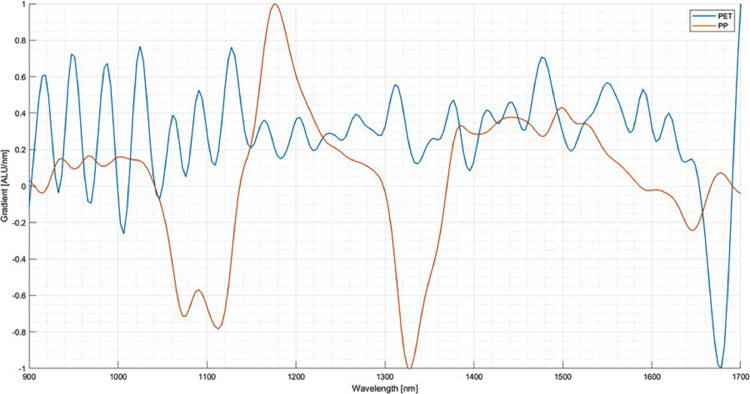
Recorded spectra using NIR technology on the experimental sensor-based sorting setup and further evaluated in MATLAB: The blue line represents the characteristic PET spectrum while red represents the characteristic PP spectrum (authors depiction).

NIR technology makes it possible to recognise different types of plastic based on specific molecule groups - in the application example PP. Fig. 12 shows the spectra of PET (blue) and PP (red) and the differences between the two materials which are used to separate them from each other. These waves are excited to vibrate by the incident radiation. The wave oscillation energy is split in the reflected and transmitted radiation so that a corresponding absorption band results in the resulting spectrum. The detected spectrum is converted into an electrical signal and processed in an associated evaluation unit. The measured spectrum is compared with several reference spectra from a database. If the spectrum matches one of these spectra, the particle is recognised as the related material and can be sorted. The detection of dark (soot-blackened) materials is a limiting factor that plays a role in plastic processing in particular. These particles usually do not reflect a spectrum detected by the NIR sensor of a sensor-based sorting machine [17].

According to the task, if packaging from the "PP" group is recognised, it must be separated from the rest of the fraction. That is done using a compressed air blast. A valve bar (6) downstream of the sensor opens one or more valves when the PP is in front of the valve bar. The PP is ejected over the separating edge (7). All other types of plastic are deliberately not ejected.

### Method validation

**Fig. 13:**
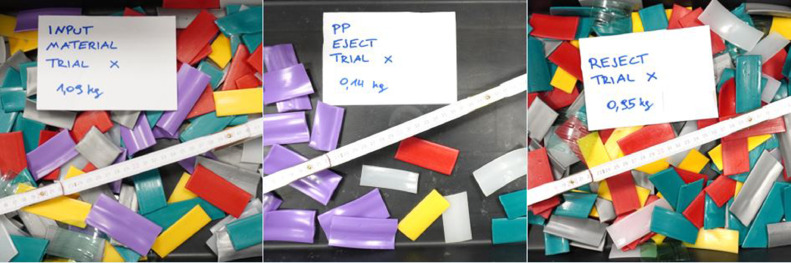
Feed material (left), separated PP – Eject (centre) and coloured plastic – Reject (right) (Trial 10 of Table 7) (authors depiction).

The validation use-case is to separate PP as target fraction from the feed material described in Table 4 and Fig. 13.

The throughput rate and the quality parameters are evaluated according to the equations shown for VIS technology. In Table 7, the data from the NIR sorting trial are summarised, where the PP material fraction was targeted for ejection. Table 8 provides the consequent results of the sorting trial in terms of plant and quality performance parameters. Both trials were performed with a different amount of input stream out of the same input fraction. The resulted fractions from the trial are shown in the centred and right picture of Fig. 13.

**Table 7:** Data of the NIR experiment.

	Unit	Trial 1	Trial 2	Trial 3	Trial 4	Trial 5	Trial 6	Trial 7	Trial 8	Trial 9	Trial 10
Time of experiment	*s*	41	41	36	38	38	40	38	40	37	38
Input mass	*kg*	1.09	1.09	1.09	1.09	1.09	1.09	1.09	1.09	1.09	1.09
Mass of eject	*kg*	0.11	0.02	0.11	0.13	0.10	0.11	0.12	0.10	0.14	0.14
Mass of reject	*kg*	0.98	0.98	0.98	0.96	1.00	0.98	0.97	0.99	0.95	0.95
Target material in eject	*kg*	0.10	0.01	0.10	0.10	0.09	0.10	0.10	0.09	0.10	0.10
Target material in reject	*kg*	0.00	0.00	0.00	0.00	0.01	0.00	0.00	0.01	0.00	0.00
Non-target material in eject	*kg*	0.01	0.01	0.01	0.03	0.01	0.01	0.02	0.01	0.04	0.04
Non-target material in reject	*kg*	0.98	0.98	0.98	0.96	0.99	0.98	0.97	0.98	0.95	0.95

**Table 8:** Results of the NIR experiment.

	Unit	Trial 1	Trial 2	Trial 3	Trial 4	Trial 5	Trial 6	Trial 7	Trial 8	Trial 9	Trial 10
Throughput-rate	kg/(h*m)	191.2	191.2	217.8	206.3	206.3	196.0	206.3	196.0	211.9	206.3
Purity	*%*	91.7	66.7	90.8	76.3	93.9	89.4	84.9	92.2	69.9	74.1
Yield	*%*	100.0	100.0	100.0	100.0	92.1	100.0	100.0	93.1	100.0	100.0
Recovery	*%*	10.0	1.4	10.0	12.0	9.1	10.4	10.9	9.4	13.1	12.4
Incorrect discharges	*%*	0.9	0.5	1.0	3.1	0.6	1.2	1.8	0.8	4.4	3.5

## Induction Sorting

### Method principle

The principal workings of induction sorting are well explained and understood. Therefore, the following will be a summary of the methods working principles [18]. Valuable metal content can be separated from the non-metallic waste stream by deploying three different methods. One of those, apart from eddy current sorting and magnetic sorting, are induction sorting systems. These sensors identify metallic objects in the waste stream via magnetic induction. Coils in the sensor generate a magnetic field, which, once a metallic object, or, in broader terms, a conductive object, moves past, it induces an electric current. According to the programming, this electronic signal is sent to a computing unit that activates an ejector mechanism, usually in the form of a compressed air nozzle array. This compressed air pushes the detected metal objects over a diverting screen, separating them from the material flow and generating a metallic fraction.

**Fig. 14:**
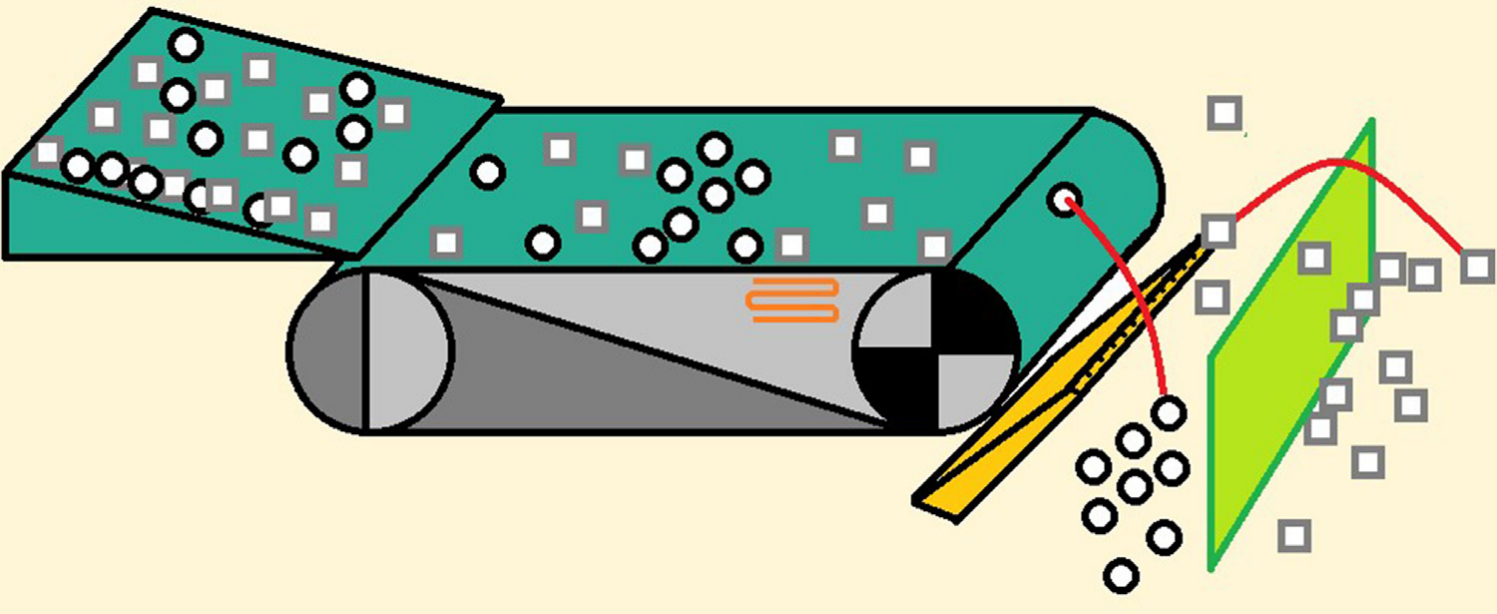
Typical working schemata of induction sorting with compressed air bar and sorting screen (authors depiction).

The size of the coils depends on the grain size of the material to be sorted and has to be chosen accordingly. Fig. 14 shows the working principle of an induction separating unit.

### Method description

In contrast to develop sorting models for the VIS and NIR sensor, the sorting model for induction consists only on the setup of parameters for the induction sensor. These parameters can be set on the man-machine-interface (MMI) of the experimental sorting setups control cabinet. These parameters are the follows:

• Delay time [ms]: Defines the time from the sensors object detection to the activation of the valve.
• Minimum blow-out time [ms]: Defines how long the valve are minimum opened.
• Minimum object size [mm]: Minimum size of an object that the valves from the air nozzle bar opens.
• Scaling [%]: Object scaling can either stretch or compress the object, it can be set from 50 to 100 %.
• Edge valve: A button to be activated, when the edge valves of the compressed air nozzle bar should be activated.
• Sensitivity: Defines the threshold value when the metal sensor should detect metal objects as metal objects, this threshold can be set from 5 to 750.

### Method application

The induction sorting system complements magnetic sorting and eddy current separation for recovering residual metals from a mix of materials. It is particularly suitable for stainless steel and composite materials such as cables or circuit boards. It can be used to focus on the production of recoverable metal concentrates, such as a stainless-steel fraction. However, the goal of processing can also be to produce a metal-free residual fraction with less than 1% metal to meet acceptable qualities and purities, e.g. in the production of residue derived fuels. Both tasks are the core applications of induction sorting systems.

Metallised foils can be separated from their unmetalled counterparts because the detection sensitivity of the induction sensor can be increased until the minute amount of metallisation can be detected. This approach allows the detection of metallised 2D materials and permits their ejection. Metallised foils are inherently difficult to be detected with a NIR sensor. There is a high probability that the metallised layer will be the side facing the NIR detector, prohibiting any form of NIR detection since the NIR inactive metal layer reflects most radiation. It is, therefore, useful to detect those metallised particles by induction sorting. Further, the reaction time between detection and ejection can be modified to account for the aerodynamics of the material. Metallised foils drop comparatively slowly, so the reaction time could be increased while sensitivity and reaction time had to be decreased when separating refuse derived fuel (RDF) from metallic contaminants.

### Method validation

**Fig. 15:**
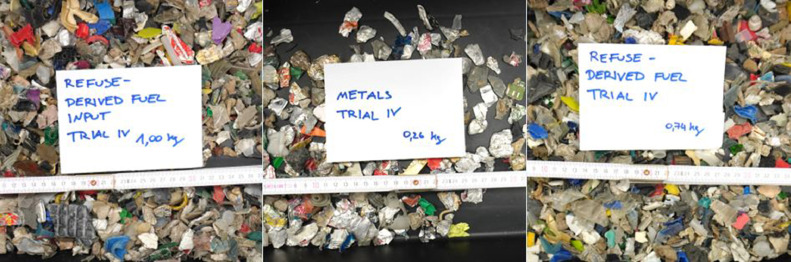
Feed material (left), separated metal – Eject (centre) and refuse-derived fuel – Reject (right) (Trial 4 of Table 9) (authors depiction).

The induction sensor settings for the following trial are a delay time of 65 ms, a minimum blow-out time of 15 ms, a minimum object size of 3 mm, a scaling of 100 %, activated edge valves and a sensitivity of 35. The throughput rate and the quality parameters are evaluated according to the equations shown for VIS technology. In Table 9, the data from the induction sorting trial are summarised, where the metals in a refuse-derived fuel stream were targeted for ejection. Table 10 provides the consequent results of the sorting trial in terms of plant and quality performance parameters. The resulted fractions from the trial are shown in the centred and right picture of Fig. 15.

**Table 9:** Data of the induction experiment.

	Unit	Trial 1	Trial 2	Trial 3	Trial 4	Trial 5	Trial 6	Trial 7	Trial 8	Trial 9	Trial 10
Time of experiment	*s*	52	53	52	55	47	53	53	49	57	55
Input mass	*kg*	1.00	1.00	1.00	1.00	1.00	1.00	1.00	1.00	1.00	1.00
Mass of eject	*kg*	0.27	0.27	0.26	0.26	0.25	0.24	0.24	0.26	0.24	0.24
Mass of reject	*kg*	0.73	0.73	0.74	0.74	0.75	0.76	0.76	0.74	0.76	0.76
Target material in eject	*kg*	0.19	0.2	0.19	0.2	0.2	0.19	0.18	0.19	0.18	0.19
Target material in reject	*kg*	0.06	0.05	0.06	0.05	0.05	0.06	0.07	0.06	0.07	0.06
Non-target material in eject	*kg*	0.08	0.07	0.07	0.06	0.05	0.05	0.06	0.07	0.06	0.05
Non-target material in reject	*kg*	0.67	0.68	0.68	0.69	0.70	0.70	0.69	0.68	0.69	0.70

**Table 10:** Results of the induction experiment.

	Unit	Trial 1	Trial 2	Trial 3	Trial 4	Trial 5	Trial 6	Trial 7	Trial 8	Trial 9	Trial 10
Throughput-rate	kg/(h*m)	138.5	135.8	138.5	130.9	153.2	135.8	135.8	146.9	126.3	130.9
Purity	*%*	70.4	74.1	73.1	76.9	80.0	79.2	75.0	73.1	75.0	79.2
Yield	*%*	76.0	80.0	76.0	80.0	80.0	76.0	72.0	76.0	72.0	76.0
Recovery	*%*	27.0	27.0	26.0	26.0	25.0	24.0	24.0	26.0	24.0	24.0
Incorrect discharges	*%*	10.7	9.3	9.3	8.0	6.7	6.7	8.0	9.3	8.0	6.7

## Sensor fusion

### Method principle and method description

In principle, all separation characteristics that can be measured without contact using sensors, such as shape, color, gloss, molecular composition, density or electrical conductivity are used. Today, various detection methods are mostly used combined to ensure simultaneous detection of multiple material properties, this is called multi-sensor technology or sensor fusion [17,19]. This approach is useful for sorting material compositions. An example is the fusion of previously described technologies NIR, VIS and induction, to eject white glass from a mixed waste fraction composed of plastics, mixed coloured glas, wire glass and metals).

Further sensor fusion techniques currently employed and under development, like X-Ray or marker-based sorting can further increase the efficiency of sensor fusion by increasing the number of physical and chemical properties and manmade markers by which sorting of refuse can be undertaken.

### Method application

**Fig. 16:**
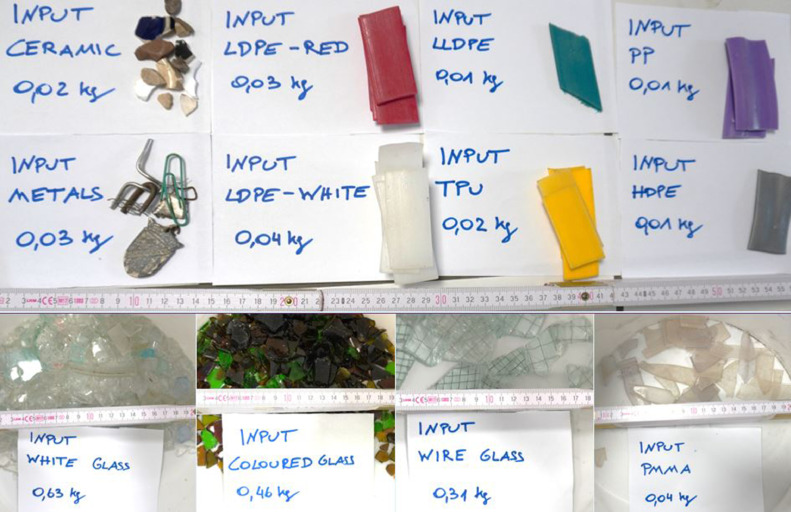
Input composition of sensor fusion trial (authors depiction).

The method described here utilises the aforementioned technologies, NIR, VIS and induction combined to generate a valuable product of pure white glass from an input consisting of LDPE, HDPE, PP, TPU, linear low density polyethylene (LLDPE), polymethylmethacrylate (PMMA), mixed coloured glass, wire glass and metals (Table 11 and Fig. 16). In one trial, NIR combined with VIS spectroscopy is used to eject only the valuable white glass by combining detection of the characteristic plastic NIR fingerprints to sort out plastics with the inclusion of the respective VIS model for white glass. Further, induction classification of the particle is set up negative, assuring the white glass fraction is not polluted by wire glass particles which would be ejected alongside the white glass. This sensor fusion ensures, that only white glass is ejected.

**Table 11:** Input composition of sensor fusion trial.

Input Material	Unit	Mass
PP	*kg*	0.01
HDPE	*kg*	0.01
TPU	*kg*	0.02
LLDPE	*kg*	0.01
LDPE - Red	*kg*	0.03
LDPE - White	*kg*	0.04
PMMA	*kg*	0.04
White Glass	*kg*	0.63
Wire Glass	*kg*	0.31
Coloured Glass	*kg*	0.46
Ceramics	*kg*	0.02
Metals	*kg*	0.03

### Method validation

**Fig. 17:**
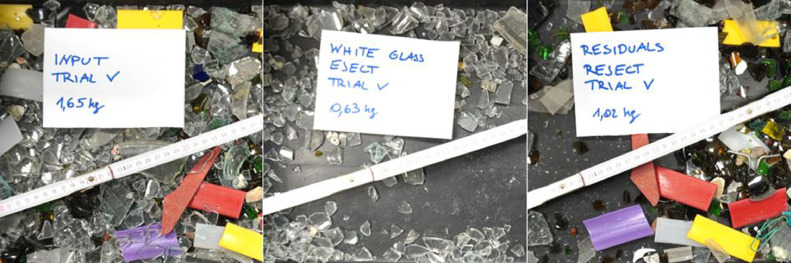
Feed material (left), separated white glass – Eject (centre) and residuals – Reject (right) (Trial 5 of Table 12) (authors depiction).

The validation use-case is to sort white glass as target fraction from the feed material described in Table 11 and Fig. 17.

The throughput rate and the quality parameters are evaluated according to the equations shown for VIS technology. In Table 12, the data from the sensor fusion trials are summarised, where the white glass was targeted for ejection. Table 13 provides the consequent results of the sorting trial in terms of plant and quality performance parameters. Both trials were performed with a different amount of input stream out of the same input fraction. The resulted fractions from the trial are shown in the centred and right picture of Fig. 17.

**Table 12:** Data of the sensor fusion experiment.

	Unit	Trial 1	Trial 2	Trial 3	Trial 4	Trial 5	Trial 6	Trial 7	Trial 8	Trial 9	Trial 10
Time of experiment	*s*	57	47	54	50	52	56	50	53	48	50
Input mass	*kg*	1.66	1.66	1.66	1.65	1.65	1.65	1.65	1.65	1.65	1.65
Mass of eject	*kg*	0.63	0.62	0.61	0.63	0.63	0.63	0.60	0.61	0.61	0.60
Mass of reject	*kg*	1.02	1.04	1.09	1.02	1.02	1.02	1.05	1.04	1.04	1.05
Target material in eject	*kg*	0.63	0.62	0.61	0.62	0.63	0.63	0.60	0.61	0.61	0.60
Target material in reject	*kg*	0.02	0.04	0.04	0.02	0.02	0.01	0.05	0.03	0.03	0.04
Non-target material in eject	*kg*	0.00	0.00	0.00	0.01	0.00	0.00	0.00	0.00	0.00	0.00
Non-target material in reject	*kg*	1.00	1.00	1.05	1.00	1.00	1.00	1.01	1.00	1.01	1.01

**Table 13:** Results of the sensor fusion experiment.

	Unit	Trial 1	Trial 2	Trial 3	Trial 4	Trial 5	Trial 6	Trial 7	Trial 8	Trial 9	Trial 10
Throughput-rate	kg/(h*m)	209.1	253.5	220.7	237.9	228.7	212.1	237.9	224.0	247.1	237.5
Purity	*%*	100.0	100.0	100.0	98.9	100.0	100.0	100.0	100.0	100.0	100.0
Yield	*%*	97.4	93.9	93.8	96.9	97.4	97.8	92.7	94.7	95.9	93.8
Recovery	*%*	38.3	37.3	36.7	38.2	38.1	38.2	36.0	36.9	37.1	36.6
Incorrect discharges	*%*	0.0	0.0	0.0	0.7	0.0	0.0	0.0	0.0	0.0	0.0

## Declaration of Competing Interest

The authors declare that they have no known competing financial interests or personal relationships that could have appeared to influence the work reported in this paper.
